# A Bleeding Kiss: intramural haematoma secondary to balloon angioplasty

**DOI:** 10.1186/1476-7120-5-21

**Published:** 2007-06-05

**Authors:** Cheerag Shirodaria, William J Van Gaal, Adrian P Banning

**Affiliations:** 1 Department of Cardiology, John Radcliffe Hospital, Oxford, UK

## Abstract

**Background:**

Intramural coronary haematoma following percutaneous coronary intervention in the absence of coronary dissection is a rare phenomenon.

**Case presentation:**

A 69 year old lady with previous prosthetic aortic valve replacement underwent percutaneous coronary intervention (PCI) from the left mainstem to the left anterior descending artery (LAD) and kissing balloon inflations to the LAD and circumflex (Cx) arteries. Although intravascular ultrasound examination (IVUS) of both the LAD and Cx showed both vessels to be widely patent at the end of the procedure, she developed ischaemic chest pain six hours later. Repeat coronary angiography revealed a significant stenosis in the proximal Cx vessel, which was confirmed on IVUS to be intramural haematoma.

**Conclusion:**

In patients taking warfarin in addition to standard antiplatelet therapy, kissing balloon inflations should be carried out with caution.

## Background

Intramural haematoma in a coronary artery is a rare complication of percutaneous coronary intervention. Haemorrhage commonly occurs due to subintimal dissection without a flap or reentry.[1,2] However, haematoma in the absence of coronary dissection with an intact media is rare.

## Case presentation

**Figure 1:**
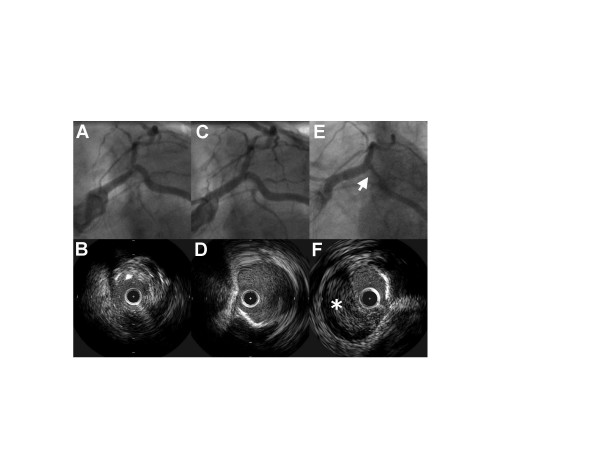
**Intramural haematoma secondary to balloon angioplasty**. *Panels A&B*: Initial angiography and IVUS examination confirmed ostial LAD disease. *Panel C*: Post procedure angiography showed a good angiographic result in the LAD. *Panel D*: IVUS examination of the Cx revealed no evidence of dissection. *Panels E&F*: Repeat angiography revealed a significant stenosis in the proximal Cx, which was confirmed on IVUS to be intramural haematoma.

A 69 year old lady with previous prosthetic aortic valve replacement and coronary bypass surgery (left internal mammary artery (LIMA) to the left anterior descending (LAD) artery) was admitted with exertional angina. Stress echocardiography had demonstrated ischemia in the LAD territory. Coronary angiography and intravascular ultrasound (IVUS) examination revealed a critical ostial stenosis in the native LAD (figure 1, panels A&B), with only a small amount of retrograde flow from the LIMA into the native LAD. IVUS examination of the circumflex (Cx) artery revealed no significant disease. A 16 mm Liberte (Boston Scientific) stent was deployed from the left mainstem (LMS) into the LAD, crossing the Cx ostium. Kissing balloon inflations were performed to the LAD and Cx, and an excellent angiographic result was achieved (figure 1, panel C). In particular, IVUS examination of the Cx at completion of the procedure showed that the vessel was widely patent, with no evidence of dissection (figure 1, panel D). Six hours after the procedure, the patient developed chest pain associated with new onset rapid atrial fibrillation (rate = 160 bpm) and ST segment depression in the lateral chest leads. Repeat coronary angiography revealed a significant stenosis in the proximal Cx vessel, which was confirmed on IVUS to be intramural haematoma (figure 1, panels E&F, see additional file 1). There was no evidence of coronary dissection, with the intima remaining intact. The LAD stent was widely patent with no evidence of haematoma. The new Cx lesion was successfully treated in a T-fashion with a 12 mm Liberte stent (Boston Scientific). Results were confirmed with IVUS.

## Discussion

Although minor haemorrhage following dissection is common, haemorrhage with an intact media is rare. Although in this case coronary dissection remains a possible cause, a number of factors suggest that this was medial/adventitial haemorrhage. First, IVUS examination of both the LAD and Cx arteries at the end of the first intervention was normal. Second, it is unusual for a dissection to localise to such a short area of coronary artery. Third, the time course of events, with symptoms only becoming apparent many hours after the procedure had finished, would be unusual for procedural coronary dissection. Indeed, the only aggravating factor in this case was that the patient was taking warfarin in addition to aspirin and clopidogrel. At the time of the initial procedure, the patient's International Normalised Ratio (INR) was 3.3. A possible mechanism in our patient was haemorrhage occurring within the aortic wall due to rupture of the vasa vasorum.

## Conclusion

In patients taking warfarin in addition to standard antiplatelet therapy, kissing balloon inflations should be carried out with caution.

## Competing interests

The author(s) declare that they have no competing interests.

All authors read and approved the final manuscript

## Supplementary Material

Additional file 1 IVUS. The image file shows the IVUS run of the circumflex artery detailing the intramural haematoma. Click here for file
